# Dietary taste patterns and diet quality of female nurses around the night shift

**DOI:** 10.1007/s00394-023-03283-w

**Published:** 2023-12-06

**Authors:** Mariëlle G. de Rijk, Jeanne H. M. de Vries, Monica Mars, Edith J. M. Feskens, Sanne Boesveldt

**Affiliations:** 1 Division of Human Nutrition and Health, Wageningen University & Research, P.O. Box 17, 6700 AA Wageningen, The Netherlands; 2 TiFN, P.O. Box 557, 6700 AN Wageningen, The Netherlands

**Keywords:** Night shift, Taste, Food preferences, Diet quality, Energy intake, Dietary patterns

## Abstract

**Purpose:**

Night shift workers are at risk of making poor food choices: e.g. sleep deprivation may lead to higher food intake with innate preferred tastes, such as sweet, savoury and fatty foods. Therefore, better insight in dietary taste patterns of night shift workers may improve the understanding of their food choices.

**Methods:**

This observational study assessed dietary taste patterns of 120 female night shift working nurses and compared them to 307 women of a reference population. Dietary intake, assessed with 24-h dietary recalls, was combined with a taste intensity database, including taste profiles of 557 foods. The contribution to the daily intake of 6 taste clusters was assessed: fat, neutral, sweet/fat, sweet/sour, salt/umami/fat and bitter.

**Results:**

*During* night shifts, nurses consumed a significantly higher energy percentage (en%) of ‘neutral’ (5.9 en%), ‘sweet/sour’ (8.1 en%) and ‘sweet/fat’ (6.5 en%) tasting foods and a lower en% of ‘fat’ (− 17.1 en%) and ‘bitter’ (− 2.1 en%) tasting foods than *outside* the night shift. They consumed a larger en% from foods with a ‘sweet/sour’ (1.9 en%) taste and a lower en% from foods with a ‘bitter’ (− 2.1 en%) taste than the reference population, irrespective of age, BMI and smoking status. A higher en% and gram% of ‘fat’ tasting foods and a higher gram% ‘fat/salt/umami’ tasting foods were associated with lower diet quality.

**Conclusion:**

Our results only partly support our hypothesis that nurses would select foods with more innate taste preferences. In addition, fat and savoury tasting foods were negatively associated with their diet quality.

**Supplementary Information:**

The online version contains supplementary material available at 10.1007/s00394-023-03283-w.

## Introduction

Working outside conventional working hours has become inevitable in the current 24-h economy. For a part of the working population this means that they regularly have to work the night shift, i.e. working more than one hour between 1:00 and 6:00 AM. In the Netherlands, around 15 percent of the working population works a night shift on a regular basis [1]. In some professions this percentage is even higher. For example, 26 percent of health care workers regularly work the night shift [1].

Working the night shift is associated with higher risks of gastrointestinal and cardiovascular diseases, several types of cancer, metabolic disorders, diabetes and overweight [2–9]. These increased risks are mainly the result of a mismatch between working a night shift and natural rhythms of activity during the day and inactivity (sleep) during the night, i.e. the circadian rhythm. This circadian rhythm is mainly affected by light and darkness and is controlled by the suprachiasmatic nucleus (SCN) located in the hypothalamus. The SCN regulates many physiological processes in a 24-h cycle such as body temperature, sleep, and appetite. By working the night shift, these circadian rhythms are disrupted and could result in sleep loss [10]. Another consequence of working the night shift is that food intake is redistributed from day to night [11–13], which could disrupt the circadian rhythm even further. Moreover, night shift workers have a tendency to have a higher meal frequency and a poorer diet quality than day shift workers [9, 14] which could be due to changes in their circadian rhythm, and could consequently contribute to heightened health risks.

Sleep-deprived people have a higher preference for sweet and savoury tastes and favour energy-rich and high-fat foods [2, 15–18], which could explain their choice for snacks instead of regular meals [19–22]. When people are sleep-deprived, they have fewer cognitive reserves to make informed decisions [19]. Consequently, decisions may be made more automatically and predominantly based on habits. This may also be the case when making food choices. Food choices in a sleep-deprived state may then revert back to strong innate taste preferences, such as those for sweet and salty/savoury foods [20, 23], taste qualities that are initially associated with nutrient and energy content of the food [23–26].

Altogether, an altered circadian rhythm together with a high risk on sleep deprivation, caused by working the night shift, could shift dietary patterns towards an unhealthy diet [9]. As sensory characteristics (flavour) are important drivers in these food choices [24, 26, 27] it is recommended to study dietary patterns not only from a nutritional perspective but also from a sensory perspective [28–30]. One way of doing this is to combine food intake data with taste characteristics of the consumed foods.

Therefore, the main aim of this study was to assess dietary taste patterns in female night shift working nurses and compare these during and outside the night shift. In addition, we compared the dietary taste patterns of the female night shift working nurses with those of a female reference population. Lastly, we studied associations between dietary taste patterns and the adherence to Dutch dietary guidelines in night shift working nurses. We hypothesized that night shift working nurses would consume more sweet and savoury fatty foods during their night shift and compared to the reference population, and have a lower adherence to the Dutch dietary guidelines. A better insight into the dietary taste patterns of night shift workers may contribute to a greater understanding of their food choices and to dietary guidelines for night shift workers.

## Methods

### Study populations

#### Night shift working nurses: Etmaal study

The observational, so-called Etmaal, study was conducted between April 2015 and July 2018 in nurses working the night shift in three hospitals located in the surrounding of Wageningen, the Netherlands [31]. In total, 164 nurses, aged 20 to 61 years, participated in this study. Nurses were included in the study when they were working the night shift for at least 6 months, were not using drugs that could cause or reduce sleep problems, were not using daylight lamps on the workplace during the night shift, were eating according to a Dutch eating pattern (2 cold meals and 1 hot meal, as—based on results from the Dutch National Food Consumption Survey—breakfast and lunch typically consist of bread-based meals while dinner is a hot meal that includes potatoes, rice or pasta), were not pregnant or breastfeeding and were not donating blood 1 week before and during the data collection period. In addition, in the current analysis we only used data of 120 female nurses that completed at least two 24-h dietary recalls. Ten males were excluded from the data analysis, as they formed a minority of the study population and could affect the study results. Male nurses, for example, had a higher energy intake, a higher variation in intake and different taste patterns than female nurses [29]. All participants provided oral and written informed consent before the start of the study. The study was approved by the medical ethical committee of Wageningen University (ABRnr: NL54414.081.15) and was conducted according to the declarations of Helsinki 2013.

## Reference population: Dutch national food consumption survey

A selected group from the most recent Dutch National Food Consumption Survey (DNFCS) 2012–2016 was used as a reference population. The DNFCS consists of a representative sample of the Dutch population regarding age, gender, region, educational level and level of urbanisation [32]. We selected 24-h dietary recall data from females, aged 20 to 61 years, with an intermediate or higher educational level to be able to have similar demographic characteristics as the night shift working nurses. We did not include data from participants when one of the 24-h dietary recalls included a special day because of fasting, illness, night work and traveling. Moreover individuals who were pregnant, breast feeding or seriously underweight were not included. In total, we included data from 307 females in the data analysis as a reference population.

### Dietary intake assessment

#### Night shift working nurses: Etmaal study

In the Etmaal study, food consumption data was self-administered by the nurses via Compl-eat, a web-based program. Compl-eat is based on a validated technique to increase the accuracy of dietary recalls [33], and includes foods that are consumed in a Dutch food pattern [34]. Nurses were asked to complete a 24-h dietary recall three times over the first day of three non-consecutive night shifts series. They reported all the foods and drinks they consumed from the evening meal prior to the night shift until the evening meal after the night shift, including the time of day. All foods and drinks reported before and after the night shift in the 24-h dietary recall were considered as food consumption ‘*outside* the night shift’.

## Reference population: Dutch national food consumption survey

In the DNFCS food consumption data was assessed via two non-consecutive 24-h dietary recalls conducted by trained interviewers [32]. The interview took place via the telephone and the interviewers used the computer-based program GloboDiet for standardisation of the 24-h dietary recalls [35]. The recalls were conducted throughout the year to cover for any seasonal effects and to represent all days of the week, including weekend days. There were at least 4 weeks between the two recalls.

### Dietary taste patterns

To assess dietary taste patterns of both the nurses and the reference population, the dietary intake data of each individual was combined with taste intensity values. Each reported food was linked to taste intensity values of the specific food derived from a taste database. The construction of the taste database is described elsewhere and the database itself can be found here [10.17026/dans-xst-ughm] [36]. Briefly, trained panellists evaluated 557 selected foods on the intensity of sweet, salt, sour, bitter, umami and fat sensation according to a modified Spectrum™ method [37]. The tested foods were selected from the Dutch National Food Consumption Survey 2007–2010 (DNFCS 2007–2010) based on consumption frequency, largest contribution to energy and macronutrients intake, and contribution to consumption variety [34, 37].

From the 557 tested foods, 468 foods were reported in the more recent DNFCS 2012–2016 from which we selected the reference population. For the 1172 foods that were not tested, but reported in this survey, a taste intensity value was estimated. This was done by using the average taste values of corresponding food groups (Suppl. Fig. S1) [32]. The same procedure was performed for the additional 141 foods, that were consumed only by the night shift working nurses. Foods that were not consumed as a single food did not receive an estimated food group average taste intensity value, because no foods were tested in these food groups, or were not frequently consumed by the Dutch population or the night shift working nurses (less than 5 times), were neglected in this analysis, e.g. foods from the food groups ‘herbs and spices’, and ‘preparations’ including medical nutrition and sport drinks. Furthermore, we recoded sugar in coffee and tea, milk in coffee, and lemonade syrup in water as these are reported as single foods in the 24-h dietary recalls, but were most often consumed in combination with the aforementioned foods and also tested by the taste panel in this combination. So finally we had taste intensity values for 1781 food codes. In total, foods responsible for 99% of energy intake in both study populations were classified into one of the taste clusters. Based on this, 6 taste clusters were identified using hierarchical cluster analyses, yielding a ‘neutral’, ‘fat’, ‘bitter’, ‘sweet and sour’, ‘fat, salt and umami’, and ‘sweet and fat’ taste cluster [29, 37–39] (Suppl. Table S1). We assessed dietary taste patterns based on the average food intake of the 24-h dietary recalls. For each individual we calculated the percentage of total energy intake and percentage of total consumed amount (in gram) for each taste cluster.

### Dutch healthy diet 2015 index

To determine diet quality for both populations, we calculated the Dutch Healthy Diet 2015 index (DHD2015-index) score based on the food and nutrient intake of the average of the two or three 24-h dietary recalls. The DHD2015-index assesses to what extent someone’s diet complies with the Dutch dietary guidelines from 2015 [40]. It consists of fifteen components: vegetables, fruit, wholegrain products, legumes, nuts, dairy, fish, tea, fats and oils, coffee, red meat, processed meat, sweetened beverages and fruit juices, alcohol and salt. For each component a maximum of 10 points can be allotted, which means complete adherence to the Dutch dietary guideline for that specific component [41]. Since the 24-h dietary recalls did not distinguish between types of coffee products, this component score was not calculated. This resulted in a total score ranging between 0 (no adherence) and 140 (complete adherence with the 2015 Dutch dietary guidelines).

### Covariates

At the start of the data collection period of the Etmaal study, nurses filled out a demographic questionnaire and their height and body weight were measured by de researchers at the working place. Height was measured without shoes using a stadiometer to the nearest 0.5 cm. Body weight was measured without shoes using an analogue weighing scale to the nearest 0.5 kg or digital weighing scale to the nearest 0.1 kg depending on the scale that was available in the hospital where they worked. For the reference population, height and body weight were self-reported by the participants during the first interview. For both populations, height and body weight were used to calculate body mass index (BMI) in kg/m^2^.

### Statistical analysis

Data was analysed using IBM SPSS Statistics 25. *p*-values below 0.05 were considered statistically significant. ANOVA and chi-squared analysis were used to compare demographic characteristics between night shift working nurses and the reference population.

Repeated measures ANOVA was used to determine differences in the percentages of energy intake and amount consumed (gram) between the taste clusters in night shift working nurses *during* the night shift and *outside* the night shift. The energy percentages of the six taste clusters were included as a dependent variable. MANCOVA was used to determine differences in the percentages of energy intake and consumed amount (gram) between the taste clusters of the night shift working nurses and the reference population. All models were adjusted for age (years), BMI (kg/m^2^), and smoking status (yes/no). Adjustment for education level did not result in a better model or different results and was therefore not included in the model. Intraclass correlation coefficients (ICC) were calculated to determine day-to-day variation of energy intake, and taste clusters between the 24-h dietary recalls of both study populations (Suppl. Table S2). Intraclass correlation coefficients (ICC) above 0.4 were considered to show fair to good reliability and an ICC above 0.75 show excellent reliability and thus less day to day variation [42].

A General Linear Model (ANCOVA) was used to determine differences in the total DHD2015-index score, adjusted for age, BMI, and smoking status, between the night shift working nurses and the reference population. Generalized Linear Models were used to determine differences in the component scores of the DHD2015-index between the night shift working nurses and the reference population. All models were adjusted for age, BMI, and smoking status. Spearman correlation coefficients were used to investigate the association between the percentage of energy intake and consumed amount (gram) of each taste clusters with the adherence to the Dutch dietary guidelines (DHD2015-index). The 95% confidence interval was calculated using Fisher’s Z-transformation.

#### Sensitivity analyses: misreporting of energy intake

We performed sensitivity analysis to examine the effects of misreporting of reported energy intake (EI) on dietary taste patterns. We identified potential low and high energy reporters by evaluating the ratio EI: Basal Metabolic Rate (BMR) at the individual level [43, 44]. BMR was estimated by the Henry equation from body weight, taking into account age and gender [45]. For the night shift working population we calculated a lower cut-off limit for the ratio of 0.97 and an upper cut-off limit of 2.46. For the reference population we calculated a lower cut-off limit of 0.93 and an upper cut-off limit of 2.57.

## Results

### Demographic characteristics

On average the night shift working nurses were slightly older than the reference population (41.4 vs. 38.2 y), their BMI was slightly lower (25.0 vs. 25.9 kg/m^2^), and a smaller proportion defined themselves as currently smoking (5.8% vs. 21.5%) (Table 1).

**Table 1:** Demographic characteristics of night shift working nurses and the Dutch reference population

	Night shift working nurses	Reference population	*p*-value^a^
	n = 120	n = 307	
Age, years mean ± SD	41.4 ± 11.9	38.2 ± 12.0	0.014
Body weight, kg mean ± SD	72.0 ± 12.6	74.4 ± 15.2	0.098
BMI, kg/m^2^ mean ± SD	25.0 ± 4.0	25.9 ± 5.2	0.066
< 18.5 (underweight), n (%)	1 (0.8)	9 (2.9)	0.197
18.5–24.9 (normal weight, n (%)	64 (53.3)	143 (46.6)	0.208
25–29.9 (overweight), n (%)	46 (38.3)	93 (30.3)	0.112
> 30.0 (obese), n (%)	9 (7.5)	62 (20.2)	0.002
Educational level^b^			0.656
Intermediate, n (%)	61 (50.8)	154 (50.2)	
High, n (%)	55 (45.8)	153 (49.8)	
Smoking status^b^			< 0.001
Current smoker, n (%)	7 (5.8)	66 (21.5)	
Non-smoker, n (%)	111 (92.5)	241 (78.5)	

^a^ANOVA for continuous variables and Chi-square for class variables

^b^4 participants had missing data on educational level or smoking status

### Dietary taste patterns *during* and *outside* the night shift

**Fig. 1:**
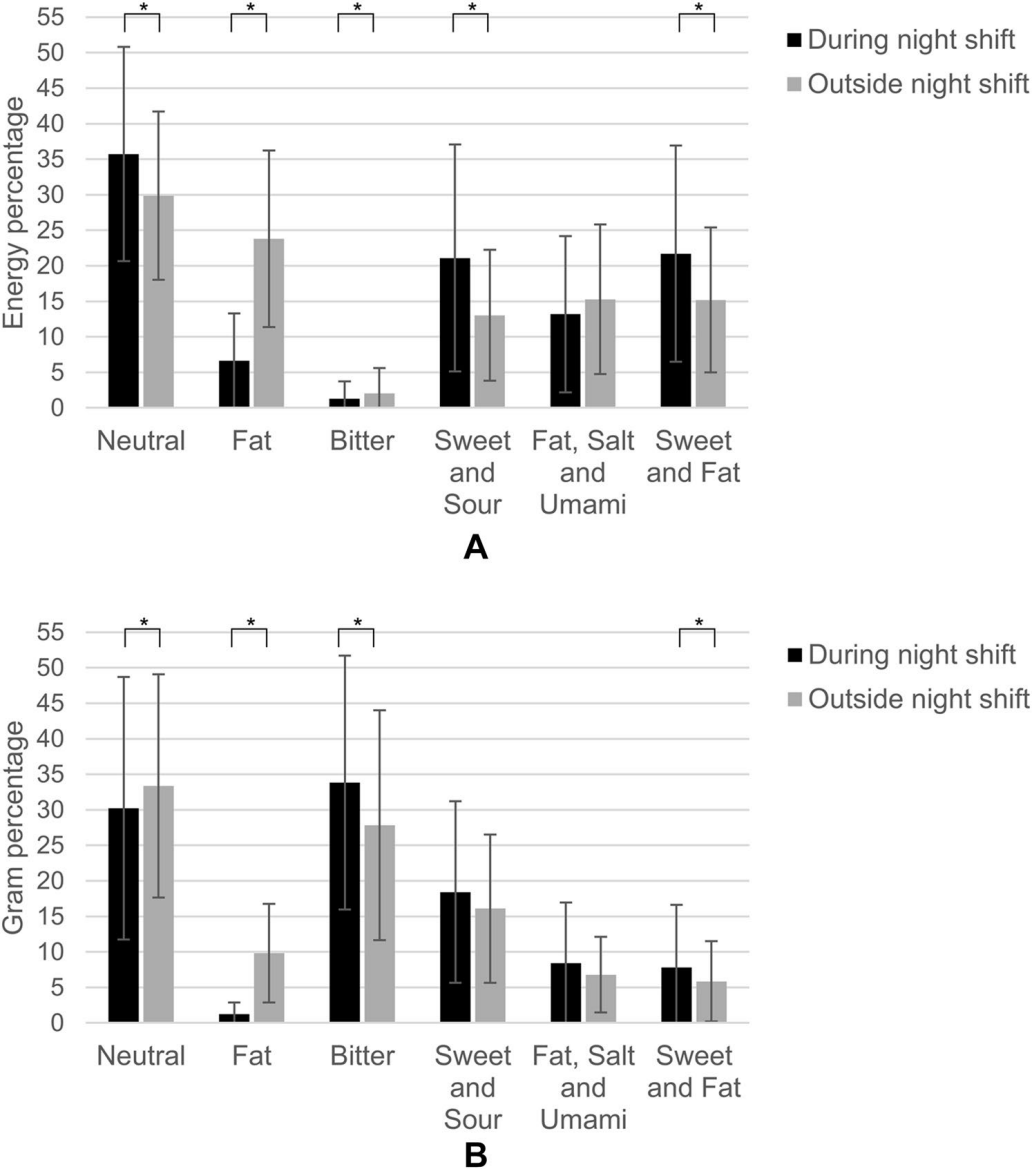
**A**, **B** Mean ± SD percentage of total daily energy intake (**A**) and total consumed amount in grams (**B**) contributed by each taste cluster *during* and *outside* a night shift (n = 120), *Significant *p* < 0.05

Different taste patterns were observed when dividing the intake assessed by the 24-h recalls between the foods consumed *during* and *outside* the night shift. When expressed in energy percentage, night shift working nurses consumed most energy from neutral tasting foods *during* (35.7 ± 15.1 en%) and *outside* (29.9 ± 11.8 en%) the night shift (Fig. 1a). Bitter tasting foods contributed least to energy intake, both *during* (1.3 ± 2.5 en%) and *outside* (2.0 ± 3.5 en%) the night shift. *During* the night shift ‘neutral’ (*p* = 0.001), ‘sweet and sour’ (*p* < 0.001) and ‘sweet and fat’ (*p* < 0.001) tasting foods significantly contributed more to energy intake and ‘fat’ (*p* < 0.001) and ‘bitter’ (*p* = 0.008) tasting foods contributed less to energy intake than *outside* the night shift.

In the neutral taste cluster, *during* the night shift most energy was derived from bread (24.9% of daily energy intake) and *outside* the night shift from bread (8.1 en%) and pasta and rice (6.7 en%). For the ‘sweet and sour’ taste cluster, fruit and yoghurt were the main contributors to energy, of which most energy from these foods were consumed *during* the night shift. Considering food choices from the ‘sweet and fat’ taste cluster, the night shift nurses consumed mainly large cookies and pastries *during* and small cookies *outside* the night shift. Regarding the ‘fat’ taste cluster *during* the night shift, the food group margarine and cooking fats was the main energy contributor, while *outside* the night shift potatoes, including fried potatoes, and margarine and cooking fats contributed most to energy.

When examining the percentage of the consumed amount (in grams) per taste clusters (Fig. 1b), the largest contribution was provided by the ‘bitter’ tasting foods coffee and tea, especially *during* the night shift (33.8 ± 17.9 g%). Also neutral tasting foods contributed a relative large amount *during* and *outside* the night shift, but the proportion was lower *during* than *outside* the night shift.

### Comparison of dietary taste patterns between night shift workers and reference population

**Fig. 2:**
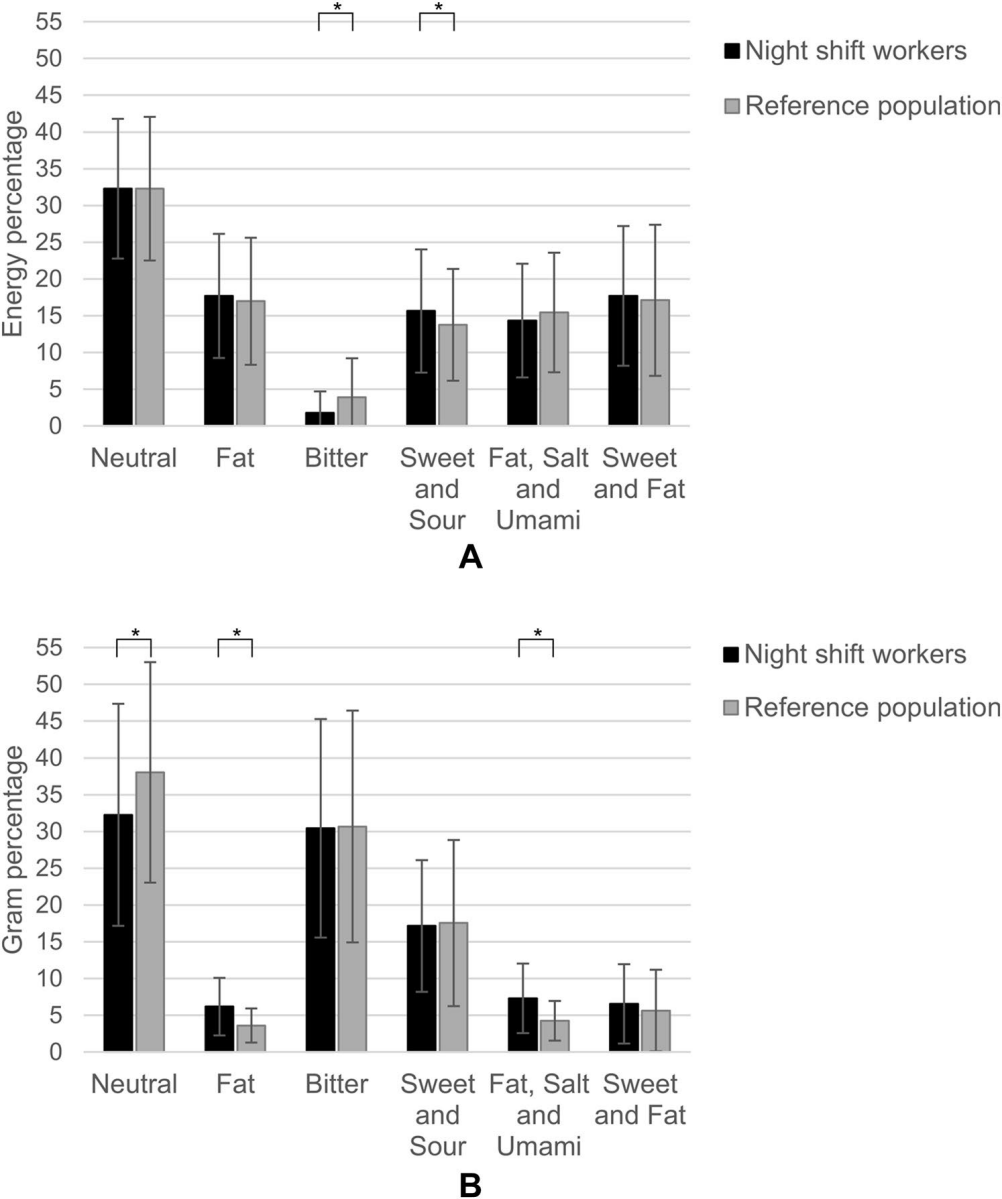
**A**, **B** Mean ± SD percentage of total daily energy intake and total consumed amount (grams) contributed by each taste cluster in night shift working nurses (n = 120) and in the reference population (n = 307). *Significant *p* < 0.05, adjusted for age, BMI and smoking

Night shift working nurses consumed on average ± SD (166 ± 55.5 kcal) less energy per 24 h than the reference population (*p* = 0.002) (Suppl. Table S2). The night shift working nurses consumed proportionally more energy from protein, carbohydrates and dietary fibre and less from fat and alcohol (0.32 ± 1.3 en%) around (*during* and *outside*) their night shift than the reference population (alcohol 1.8 ± 3.8 en%). Similar to the night shift workers, the reference population consumed the highest energy percentage from neutral tasting foods (32.3 ± 9.5 en% and 32.3 ± 9.8 en%, respectively) and the lowest energy percentage from bitter tasting foods (1.8 ± 2.9 en% and 3.9 ± 5.3 en%) (Fig. 2a). The energy contribution of foods with a ‘bitter’ taste was significantly lower in the nurses than the reference population (*p* = 0.030). In the night shift working population this energy mainly came from coffee (with milk), while in the reference population this energy mainly came from wine. Night shift workers also tended to consume a higher energy percentage of foods with a ‘sweet and sour’ taste (mainly from fruit and yoghurt) than the reference population (mainly from fruit and soft drinks; *p* = 0.05). Compared to the reference population, the night shift nurses consumed similar amounts of energy from the other taste clusters.

When examining the percentage of the consumed amount (in grams) per taste cluster (Fig. 2b); both night shift workers and the reference population consumed proportionally the largest amount of ‘neutral’ (32.2 ± 15.1 and 38.0 ± 15.0 g%) and ‘bitter’ (30.4 ± 14.9 and 30.7 ± 15.8 g%) tasting foods. Night shift working nurses proportionally consumed a significantly lower amount of ‘neutral’ tasting foods and a significantly higher amount of ‘fat’ and ‘fat, salt and umami’ tasting food than the reference population. The percentage of the consumed amount contributed by the “sweet and fat” cluster was similar.

#### Misreporting of energy intake

From the night shift working population, 26.7% of the participants was identified as potential low energy reporter, while in the reference population this was 17.9%. When excluding these low energy reporters the difference between percentage of total daily energy intake of ‘sweet and sour’ tasting foods between the night shift workers and the reference population disappeared (Suppl. Table S3). In addition, the difference in the percentage of consumed amount (in grams) of ‘neutral’ tasting foods between the night shift workers and the reference population was attenuated (*p* = 0.077) (Suppl. Table S3).

### Adherence to Dutch dietary guidelines

The overall DHD2015-index score of the two populations was similar (*p* = 0.720); night shift workers scored on average 77 ± 15 points and the reference population scored on average 74 ± 16 points (Table 2). However, there were differences in the scores of the individual components. The night shift working nurses scored significantly higher on the components ‘fruit’, ‘whole grain products’, ‘nuts’, ‘sugar sweetened beverages’ and ‘alcohol’ and significantly lower on the components ‘tea’ and ‘fat and oils’ compared to the reference population.

**Table 2:** Mean scores and standard deviations of the total score of the Dutch Healthy Diet 2015 index (DHD2015-index) and its component scores, reflecting the adherence to Dutch dietary guidelines of 120 night shift working nurses and compared with the reference population

DHD2015-component score	Night shift workers		Reference population		*p*-value^b^
	n = 120		n = 307		
	Mean	SD	Mean	SD	
Vegetables	6.9	2.7	6.3	3.2	0.186
Fruit	7.3	3.4	5.2	3.9	< 0.001
Whole grain products	5.3	2.7	4.5	2.9	0.044
Legumes	1.4	3.4	0.9	2.8	0.163
Nuts	2.0	3.6	1.1	2.9	0.033
Dairy	6.3	3.2	6.0	3.4	0.802
Fish	1.3	2.9	1.6	3.2	0.387
Tea	4.7	4.0	6.5	4.1	< 0.001
Fats and oils	3.3	4.1	6.6	4.4	< 0.001
Red meat	9.0	2.5	9.2	2.1	0.386
Processed meat	4.6	3.9	4.7	4.0	0.767
Sugar sweetened beverages	7.4	3.4	5.3	4.3	< 0.001
Alcohol	9.9	0.9	8.5	3.3	< 0.001
Sodium	7.5	2.9	7.9	2.6	0.106
Overall DHD2015-index score^a^	77.0	14.9	74.2	15.8	0.720

^a^The score ranges between 0 and 140 points

^b^Generalized Linear Models were performed to compare DHD2015-index component scores between night shift workers and the reference population, General Linear Model was used to compere the total DHD2015-index score. All models were adjusted for age, BMI and smoking

Night shift workers scored highest on the components ‘alcohol’ (9.9 ± 0.9 points) and ‘red meat’ (9.0 ± 2.5 points), meaning that they almost completely adhere to the Dutch dietary guidelines for these components. The lowest adherence scores were found for ‘fish’ (1.3 ± 2.9 points) and ‘legumes’ (1.4 ± 3.4 points).

### Associations between dietary taste patterns and adherence to Dutch dietary guidelines

The proportional intake of energy from ‘fat’ tasting foods was negatively associated with the DHD2015-index score (r = − 0.31, *p* < 0.001) (Table 3) in night shift working nurses. This means that a higher energy intake from ‘fat’ tasting foods was associated with a lower adherence—and thus a lower diet quality—to the Dutch dietary guidelines.

**Table 3:** Spearman correlation coefficients between the DHD2015-index score and the percentage consumed energy and amount from 6 taste clusters among night shift working nurses (n = 120)

Taste cluster	% Energy			% Amount (gram)		
	r	95% CI	*p*-value	r	95% CI	*p*-value
Neutral	0.15	(− 0.02, 0.31)	0.097	0.08	(− 0.12, 0.26)	0.412
Fat	− 0.31	(− 0.47, − 0.13)	< 0.001	− 0.36	(− 0.51, − 0.19)	< 0.001
Bitter	0.07	(− 0.13, 0.26)	0.477	0.24	(0.04, 0.42)	0.008
Sweet and Sour	0.16	(− 0.03, 0.35)	0.073	− 0.06	(− 0.25, 0.14)	0.544
Fat, Salt and Umami	− 0.02	(− 0.19, 0.16)	0.836	− 0.28	(− 0.44, − 0.11)	0.002
Sweet and Fat	0.04	(− 0.14, 0.23)	0.640	− 0.04	(− 0.24, 0.15)	0.641

The proportional amount of foods from the ‘fat’ (r = − 0.36, *p* < 0.001) and ‘fat, salt and umami’ (r = − 0.28, *p* = 0.002) clusters were also negatively associated with the DHD2015-index score. The proportional amount of foods from the ‘bitter’ tasting foods (r = 0.24, *p* = 0.008) was, on the other hand, positively associated with DHD2015-index score (Table 3).

## Discussion

This study assessed dietary taste patterns of female night shift working nurses and compared these *during* the night shift and *outside* the night shift. Moreover, these dietary taste patterns were compared with those of a female reference population. Last dietary taste patterns were associated with the diet quality of the nurses.

In light of our hypothesis, some of our results were inconclusive. As hypothesized we observed a higher contribution of energy from the ‘sweet and fat’ and ‘sweet and sour’ taste cluster *during* the night shift than *outside* the night shift, however there were no differences for the ‘fat, salt and umami’ taste cluster. This higher energy intake from the ‘sweet and sour’ taste cluster *during* the night shift was also reflected by a higher total daily energy intake from the ‘sweet and sour’ taste cluster in night shift working nurses than the reference population. However, we did not observe differences in energy intake from the ‘sweet and fat’ and ‘fat, salt and umami’ taste cluster between the night shift working nurses and the reference population. Also, we did not find a worse diet quality in the nurses as compared to the reference population. However, nurses with a higher energy and gram intake from ‘fat’ tasting foods and a higher gram intake of ‘fat, salt and umami’ tasting foods had a lower diet quality. On the other hand, a higher gram intake of ‘bitter’ tasting foods was associated with a better diet quality in night shift working nurses.

We expected that especially the energy intake from ‘sweet and fat’ tasting foods would be higher in night shift working nurses than in the reference population. This would be due to lower cognitive reserves and therefore a higher innate preference for sweet foods [19, 20, 23], and because sleep restriction might lead to a preference for fat foods [16]. In the current study, we indeed found that night shift working nurses consumed more energy from the ‘sweet and fat’ taste cluster *during* the night shift than *outside* the night shift. However, this higher energy intake *during* the night shift did not result in a higher total daily energy intake from the ‘sweet and fat’ taste cluster than the reference population. Nonetheless, we found that nurses had a higher intake of the ‘sweet and sour’ taste cluster, not only *during* the night shift but also compared to the reference population. To explain these inconsistent results it is important to realize that food choices are not only made on the basis of appetite or hedonic (taste) values, but are also based on the nutritional value of the food and health goals which requires cognitive control [24]. Another important factor is the availability of foods. Some companies offer meals during the night shift on the worksite. However, the meal provision in the participating hospitals was limited and therefore foods were probably taken from home. Consequently, the nurses were making their food choices at home when they were less tired and able to make well informed, healthier food choices than during the night shift. Indeed *during* the night shift mainly healthy foods were eaten such as bread, fruit and yoghurt. Thus, our results may become clearer if in future research other determinants of food choice and intake are taken into account.

The explanation that selected foods of the nurses foods were taken from home may also explain why we observed no differences between the intake of ‘fat, salt and umami’ tasting foods *during* and *outside* the night shift and as compared to the reference population. Another explanation may be that our study was performed in a female study population. Earlier research showed that females had a lower preference for savoury tasting foods (than men), making it unlikely that they would increase their intake of these foods *during* the night shift [29].

It may seem surprising that the gram percentage of ‘bitter’ tasting foods was higher *during* the night shift than *outside* the night shift, while the energy percentage was, as expected, lower *during* the night shift than *outside* the night shift. Typically, bitter is not a preferable taste and therefore you would expect that ‘bitter’ tasting foods do not contribute a lot to total energy intake and consumed amount during a night shift [24]. However, *outside* the night shift, the second largest source after coffee (with milk), of energy from this ‘bitter’ taste cluster comes from alcohol intake which has a considerably higher energy content than coffee and tea (as typically most consumed bitter drinks), while *during* the night shift no alcohol was consumed. This is also reflected in a higher energy percentage by alcohol in the refence population. On the other hand, the higher gram percentage from ‘bitter’ tasting foods *during* the night shift than *outside* the night shift may be explained by the fact that bitter tasting foods such as coffee and tea contain caffeine, which is known for its stimulating effects on the brain [46, 47], and therefore a tempting food for nurses working the night shift.

Within the neutral taste cluster there was also a discrepancy between energy and gram percentage of neutral tasting foods. Most energy and gram percentage from the neutral taste cluster *outside* the night shift came from bread, pasta/rice, milk and vegetables typically eating during the evening dinner, while a considerable energy percentage *during* the night shift was contributed by bread. While vegetables contribute most to gram percentage but not to energy percentage, for bread it is the other way around.

Diet quality of the night shift nurses was with a DHD2015-index score of 76.2 ± 14.5-comparable to that of the reference population (74.2 ± 15.8). Nurses ate more fruit and vegetables and drank less sweetened beverages and alcohol than the reference population. This was in line with the results of the dietary taste patterns in this current study. Fruit was, together with yogurt, the main source of energy intake from the ‘sweet and sour’ taste cluster, and alcohol was one of the energy sources in the reference population that contributed to a higher energy intake from the ‘bitter’ taste cluster compared to the night shift working nurses. Yet a higher energy intake from this ‘sweet and sour’ taste cluster was not associated with a better diet quality. Probably because this taste cluster also includes soft drinks and some sweet and sour sauces which are not part of a healthy diet and thus do not contribute to a better diet quality [41]. A higher gram percentage from the ‘bitter’ taste cluster was however associated with a better diet quality. This is in contrast with previous studies which showed a negative association between bitter tasting foods and a healthy and sustainable diet [48, 49]. In the previous studies, energy intake from bitter tasting foods mainly came from alcohol which is negatively associated with several health outcomes [41]. However, as mentioned before, the energy and gram percentage of bitter tasting foods of the night shift working nurses did not come from alcohol which explains why there was not an inverse correlation.

We also showed that energy and gram percentage of the ‘fat’ cluster and gram percentage of the ‘fat, salt and umami’ taste cluster were negatively associated with diet quality. This is partly in line with other studies that found that a higher energy intake of ‘fat, salt and umami’ tasting foods and a higher gram intake of ‘fat’ tasting foods was associated with a lower diet quality in the Dutch population [48, 49]. A healthy diet has been associated with less ‘fat, salt and umami’ tasting foods and more neutral taste foods than an unhealthy diet [48], where as a ‘tasteful’ diet is related to a lower diet quality. This is further reflected in an American population where the importance of taste was negatively correlated with diet quality [50].

This is the first study that investigated differences in taste patterns of nurses inside and outside the nightshift, and compared taste patterns and diet quality with a reference population. Using a taste database in addition to a diet composition database gives an extra opportunity to not only investigate the diet of night shift working nurses from a nutrient and diet quality perspective, but also from a sensory perspective. Assessing dietary taste patterns gives a better understanding of potential drivers of food choice and intake in night shift working nurses. Given that intake data is the result of foods selected (and subsequently consumed) by the nurses, any relationship of taste (intensity) with intake must be the result of its impact on food preference and choice. This information can be used in formulation of nutrition strategies for night shift workers to improve their nutritional status and consequently promoting health and wellbeing [51, 52]. A nutritional strategy could be recommending foods *during* the night shift that are perceived as ‘sweet and fat’, but are healthier than large cookies and pastries, for example a portion of low fat yoghurt with fruit. Since these foods were already consumed by some of the nurses, this could be a viable strategy for all.

A previous study by van Langeveld already showed that a taste database in combination with two 24-h dietary recalls can be used to assess dietary taste patterns [29]. In this study we also used multiple dietary 24-h dietary recalls per individual. While the total energy intake over the multiple 24-h dietary recalls did not show much day to day variation, the energy percentage from the different taste clusters over the multiple 24-h dietary recalls did. The Intraclass Correlations Coefficients (ICC) ranged from 0.10 for ‘fat’ taste cluster to 0.70 for ‘sweet and sour’ taste cluster in the night shift working population and from 0.14 for ‘fat’ to 0.54 for the ‘bitter’ taste cluster in the reference population. This means that especially energy intake from the ‘fat’ taste cluster varied greatly from day to day in both study populations, while the energy intake from the ‘sweet and sour’ taste cluster in the night shift working population was less prone to day to day variation. As we used average dietary taste patterns per person and we only compared dietary taste patterns at the group level this day to day variations was nullified [53].

We allocated the foods to the taste clusters by using hierarchical cluster analyses based on the (average) taste intensity values of each food. Although the allocation of the taste clusters to foods was done in an objective and repeatable manner, the actual taste intensity values of an untested food may deviate from the average taste intensity values it was assigned to and therefore did not end up in the correct taste cluster. However, the deviations in the assigned taste intensity values compared to the actual taste intensity values may be expected to be random, and therefore probably did not affect the current results, because we looked at dietary taste patterns at the group level and not at an individual level. Random errors may be expected to cancel out if the population is large enough.

Another methodological constraint is that nightshift working nurses were underreporting their daily energy intake. This is a well-known issue when assessing dietary intake [42, 44], but could be more prevalent in the current population due to night shift related fatigue. However, excluding potential under reporters from the analysis did not result in different dietary taste patterns.

In general, a positive energy balance will result in overweight. Although the night shift workers in our study tended to have a lower BMI than the reference population, it is more common that shift workers have a higher BMI. It is perhaps surprising that this may not necessarily result from a higher energy intake, as previous studies have shown similar energy intakes among day and night shift workers [2]. It has been shown that meal timing also could have metabolic consequences for the development of overweight and for weight loss [54]. Eating the same amount of energy earlier in the day resulted in a lower body weight than eating the same amount of energy later during the day, which is the case for night shift workers [54, 55]. In addition, the human body processes foods differently during the night than during the day. Glucose and fat metabolism are following a circadian rhythm and are disrupted by food intake during the night shift resulting in higher glucose spikes during the night than during the day [56–58]. Snacks most likely contain more sugar and fat resulting in even higher spikes. This could result in a higher disruption of the circadian rhythm and consequently higher health risks. With this observational study we aimed to assess taste patterns in night shift workers and whether a higher preference and choice of sweet and savoury tasting foods could explain the snack tendency and eventually the higher risk of having overweight.

Ideally, we would have compared dietary taste patterns around a night shift as well as around a day shift in the same population. Unfortunately, this data was not available and the current reference population is assumed to be a good alternative, although one could speculate that night shift working nurses eat healthier during a day shift than during a night shift and than the current reference population. It should also be noted that what the nurses ate outside their night shift, might still be affected by the circadian misalignment and sleep disturbance from being on the shift work schedule, and may not reflect their ‘normal day shift’ eating pattern.

The dietary intake data from night shift working nurses was only assessed during the first night shift. It is hypothesized that night shift workers suffer mostly from sleep loss during this first night shift as they prolong their day and make food choices more automatically and based on habits due to fewer cognitive reserves [19]. Potential differences in dietary taste patterns between night shift workers and the reference population would therefore be expected to be most pronounced with dietary taste patterns of night shift workers around the first night shift. However, one study showed that the energy intake of night shift workers is highest during the second consecutive night shift; nevertheless fat intake was the highest during the first night shift [13]. Dietary taste patterns could therefore be different across consecutive night shifts. Thus, we must be careful in extrapolation of our results for a first night shift to consecutive night shifts.

Lastly, our study population consisted of nurses, who might be more health-conscious than other night shift workers. Specifically, the night shift working nurses in this study consisted solely of women, and might be more interested in the role of nutrition than those who did not participate. This could explain the higher fruit and vegetable intake but also the higher energy percentage from ‘sweet and sour’ foods compared to the reference population. Thus, our results may not generalise to all nurses and especially not to other night shift working populations. Males, for example, differ in their dietary taste patterns and consume more foods with a ‘fat, salt and umami’ and ‘bitter’ taste and less foods with a ‘sweet and sour’ and ‘sweet and fat’ taste than women [29]. It would be interesting to assess dietary taste patterns in other night shift working populations especially in those with other shift work rotations or an unhealthier diet than in our population.

In conclusion, diet quality and taste patterns seem to be related in night shift working nurses. Also, our findings suggest differences between the dietary taste patterns of night shift working nurses and populations without nightshifts and within the nurses between taste patterns during and outside the nightshift. However, not all findings were consistent with our hypothesis that the nurses would select foods with more of the innate taste preferences of sweet and savoury. For example, a possible taste preference for sweet in night shift working nurses was not reflected by a higher intake of ‘sweet and fat’ tasting foods over 24 h. This also indicates that other factors, such as availability, are involved in the food choice of night shift working nurses. Lastly, we showed that a higher intake from ‘fat’ and ‘fat, salt and umami’ tasting foods were associated with a lower diet quality. Therefore, to improve diet quality in night shift working nurses, it seems to be important to include their taste preferences in nutrition strategies. Whether this finding is also true for other night shift working populations must be explored in future research.

### Supplementary Information

Below is the link to the electronic supplementary material.

Supplementary file 1 (TIF 12316 KB)

Supplementary file 2 (DOCX 23 KB)

## Data Availability

The database itself can be found here [10.17026/dans-xst-ughm] [36]. Other data can be made available upon request. The lead author had full access to the data reported in the manuscript.
