# Correction to: Dietary taste patterns and diet quality of female nurses around the night shift

**DOI:** 10.1007/s00394-023-03316-4

**Published:** 2024-01-06

**Authors:** Mariëlle G. de Rijk, Jeanne H. M. de Vries, Monica Mars, Edith J. M. Feskens, Sanne Boesveldt

**Affiliations:** 1 Division of Human Nutrition and Health, Wageningen University & Research, P.O. Box 17, 6700 AA Wageningen, The Netherlands; 2 TiFN, P.O. Box 557, 6700 AN Wageningen, The Netherlands

**Correction to: European Journal of Nutrition** 10.1007/s00394-023-03283-w

The article Dietary taste patterns and diet quality of female nurses around the night shift, written by Mariëlle G. de Rijk, Jeanne H. M. de Vries, Monica Mars, Edith J. M. Feskens and Sanne Boesveldt was originally published electronically on the publisher’s internet portal on 06 December 2023 without open access. With the author(s)’ decision to opt for Open Choice the copyright of the article changed on 19 December 2023 to © The Authors 2023 and this article is licensed under a Creative Commons Attribution 4.0 International License, which permits use, sharing, adaptation, distribution and reproduction in any medium or format, as long as you give appropriate credit to the original author(s) and the source, provide a link to the Creative Commons licence, and indicate if changes were made. The images or other third party material in this article are included in the article’s Creative Commons licence, unless indicated otherwise in a credit line to the material. If material is not included in the article’s Creative Commons licence and your intended use is not permitted by statutory regulation or exceeds the permitted use, you will need to obtain permission directly from the copyright holder. To view a copy of this licence, visit http://creativecommons.org/licenses/by/4.0/.

The original article has been corrected.

