# Supplementary figures and images for: Dietary taste patterns and diet quality of female nurses around the night shift
Source: Eur J Nutr. 2023 Dec 6;63(2):513–24. doi: 10.1007/s00394-023-03283-w (PMC10899307; doi:10.1007/s00394-023-03283-w)

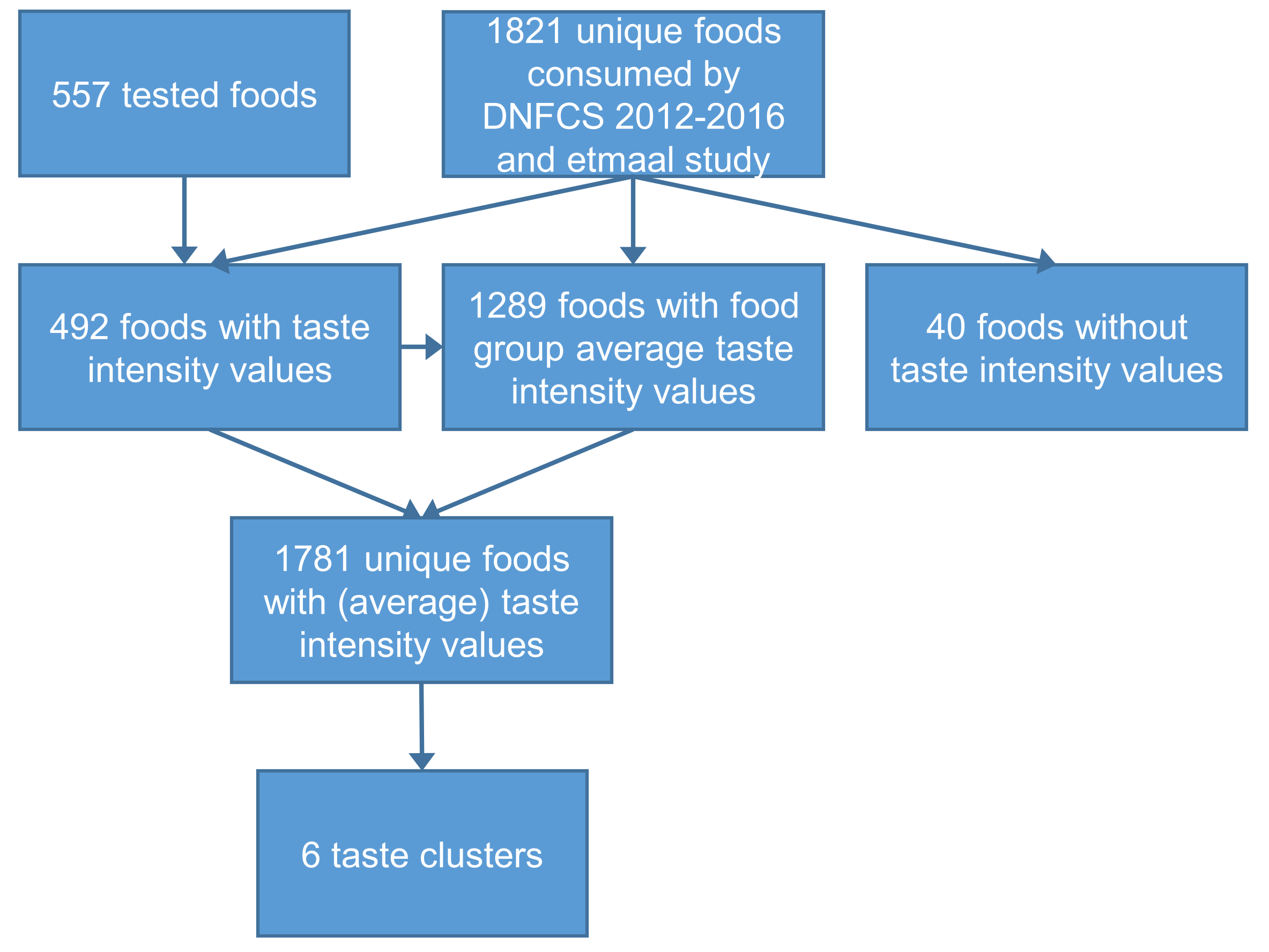

Supplement: Supplementary file 1 — (TIF 12316 KB) [file 394_2023_3283_MOESM1_ESM.tif]
