# Supplementary material for: Dietary taste patterns and diet quality of female nurses around the night shift
Source: Eur J Nutr. 2023 Dec 6;63(2):513–24. doi: 10.1007/s00394-023-03283-w (PMC10899307; doi:10.1007/s00394-023-03283-w)
Supplement: Supplementary file 2 — (DOCX 23 KB) [file 394_2023_3283_MOESM2_ESM.docx]

**Supplementary Table 1.** Taste intensity values (mean ±SD) of the 6 taste modalities and energy and nutrient content stratified by taste clusters calculated from the foods (n=1,781) consumed in the Etmaal study and by adults in the DNFCS* 2012-2016.

|  | **Neutral** | | **Fat** | | **Bitter** | | **Sweet and Sour** | | **Fat, Salt and Umami** | | **Sweet and Fat** | |
| --- | --- | --- | --- | --- | --- | --- | --- | --- | --- | --- | --- | --- |
|  | n= 376 | | n= 275 | | n= 68 | | n= 323 | | n= 365 | | n= 374 | |
| **Taste modality** | Mean | SD | Mean | SD | Mean | SD | Mean | SD | Mean | SD | Mean | SD |
| Sweet | 8.9 | 3.9 | 7.5 | 6.2 | 13.9 | 7.0 | 31.8 | 11.8 | 9.8 | 5.8 | 45.3 | 10.7 |
| Sour | 4.2 | 4.0 | 4.2 | 2.8 | 15.6 | 12.1 | 31.4 | 9.9 | 12.4 | 6.2 | 3.4 | 2.8 |
| Bitter | 3.2 | 2.7 | 1.3 | 1.4 | 34.7 | 13.8 | 2.3 | 1.6 | 2.2 | 2.6 | 3.1 | 3.8 |
| Umami | 4.1 | 2.7 | 11.6 | 1.4 | 1.3 | 13.8 | 2.5 | 1.6 | 23.6 | 2.6 | 0.8 | 3.8 |
| Salt | 9.6 | 5.4 | 24.0 | 10.7 | 2.1 | 0.9 | 6.8 | 9.6 | 42.9 | 7.0 | 8.4 | 2.9 |
| Fat sensation | 12.5 | 8.5 | 46.1 | 25.6 | 4.5 | 3.1 | 19.7 | 21.0 | 49.6 | 12.7 | 32.8 | 15.2 |
| **Nutrient content / 100g** |  |  |  |  |  |  |  |  |  |  |  |  |
| Energy (kcal) | 191 | 179 | 329 | 248 | 123 | 124 | 110 | 124 | 248 | 134 | 335 | 152 |
| Protein (g) | 6.9 | 6.1 | 10.6 | 9.7 | 1.3 | 3.3 | 1.7 | 2.5 | 13.1 | 9.8 | 4.9 | 3.6 |
| Fat (g) | 6.5 | 14.3 | 25.9 | 30.7 | 1.7 | 6.2 | 5.5 | 12.6 | 17.7 | 15.0 | 11.8 | 10.8 |
| Carbohydrates (g) | 24.0 | 24.0 | 13.1 | 21.5 | 9.3 | 13.6 | 12.7 | 15.0 | 8.8 | 14.3 | 50.7 | 25.7 |
| Mono- and disaccharides (g) | 3.7 | 6.1 | 1.7 | 5.7 | 6.8 | 9.9 | 11.3 | 13.4 | 3.1 | 7.1 | 33.2 | 22.5 |
| Dietary fibre (g) | 4.4 | 4.9 | 1.3 | 2.1 | 0.9 | 2.7 | 1.0 | 2.1 | 0.8 | 2.0 | 2.4 | 3.2 |
| Alcohol (g) | 0.0 | 0.0 | 0.0 | 0.0 | 10.1 | 11.8 | 0.0 | 0.0 | 0.0 | 0.1 | 0.0 | 0.1 |
| Sodium (mg) | 200 | 294 | 265 | 413 | 22 | 64 | 105 | 209 | 1006 | 1983 | 146 | 138 |

*Dutch National Food Consumption Survey

**Supplementary Table 2.** Mean total energy intake, macronutrient intake as energy intake percentages (en%) and en% per taste cluster of night shift working nurses and reference population with intraclass correlation coefficients (ICC) and 95% confidence intervals.

|  | Night shift workers  n=120 | | | |  | Reference population  n=307 | | | |
| --- | --- | --- | --- | --- | --- | --- | --- | --- | --- |
|  | Mean | SD | ICC* | 95% CI |  | Mean | SD | ICC | 95% CI |
| *Nutrient content* |  |  |  |  |  |  |  |  |  |
| Energy intake, kcal | 1690 | 471 | 0.67 | (0.54, 0.77) |  | 1856 | 532 | 0.62 | (0.54, 0.69) |
| Protein, en% | 16.9 | 3.6 | 0.68 | (0.56, 0.77) |  | 15.8 | 3.6 | 0.58 | (0.49, 0.65) |
| Fat, en% | 32.9 | 6.9 | 0.60 | (0.45, 0.72) |  | 35.2 | 7.0 | 0.41 | (0.28, 0.51) |
| Saturated fatty acids, en% | 12.9 | 3.5 | 0.59 | (0.43, 0.71) |  | 12.9 | 3.4 | 0.44 | (0.33, 0.54) |
| Carbohydrates, en% | 46.7 | 7.3 | 0.67 | (0.55, 0.77) |  | 44.6 | 7.8 | 0.49 | (0.39, 0.58) |
| *Taste clusters* |  |  |  |  |  |  |  |  |  |
| Neutral, en% | 32.3 | 9.5 | 0.34 | (0.08, 0.53) |  | 32.3 | 9.8 | 0.45 | (0.31, 0.56) |
| Fat, en% | 17.7 | 8.4 | 0.10 | (-0.25, 0.36) |  | 17.0 | 8.6 | 0.14 | (-0.07, 0.31) |
| Bitter, en% | 1.8 | 2.9 | 0.68 | (0.55, 0.77) |  | 3.9 | 5.3 | 0.54 | (0.43, 0.63) |
| Sweet & sour, en% | 15.6 | 8.4 | 0.70 | (0.58, 0.79) |  | 13.7 | 7.6 | 0.48 | (0.35, 0.58) |
| Fat, salt and umami, en% | 14.3 | 7.7 | 0.38 | (0.14, 0.56) |  | 15.4 | 8.1 | 0.40 | (0.24, 0.52) |
| Sweet and fat, en% | 17.7 | 9.5 | 0.67 | (0.54, 0.77) |  | 17.1 | 10.3 | 0.48 | (0.35, 0.59) |

*based on three dietary recalls in 103 night shift working nurses.

**Supplementary Table 3.** Percentage of total daily energy intake and total consumed amount (gram) contributed by each taste cluster in night shift working nurses (n=87) and in the reference population (n=252), potential low energy reporters are excluded.

|  | Night shift workers | | Reference population | |  |
| --- | --- | --- | --- | --- | --- |
|  | Mean | SD | Mean | SD | p-value* |
| Neutral, en% | 31.6 | 9.5 | 31.4 | 9.1 | 0.239 |
| Fat, en% | 18.2 | 7.5 | 17.4 | 8.4 | 0.662 |
| Bitter, en% | 1.7 | 2.8 | 4.2 | 5.4 | <0.001 |
| Sweet & sour, en% | 14.4 | 7.6 | 13.8 | 7.2 | 0.813 |
| Fat, salt and umami, en% | 14.8 | 7.2 | 15.3 | 8.1 | 0.855 |
| Sweet and fat, en% | 18.8 | 8.6 | 17.5 | 9.7 | 0.917 |
|  |  |  |  |  |  |
| Neutral, gram% | 31.8 | 14.7 | 36.5 | 14.2 | 0.077 |
| Fat, gram% | 6.7 | 3.8 | 3.8 | 2.4 | <0.001 |
| Bitter, gram% | 29.9 | 13.6 | 31.4 | 15.0 | 0.125 |
| Sweet & sour, gram% | 16.9 | 9.1 | 17.9 | 11.0 | 0.577 |
| Fat, salt and umami, gram% | 7.3 | 4.4 | 4.3 | 2.7 | <0.001 |
| Sweet and fat, gram% | 7.2 | 5.2 | 6.0 | 5.4 | 0.188 |

*Adjusted for age, BMI and smoking.
